# Peer-assessment ability of trainees in clinical restorative dentistry: can it be fostered?

**DOI:** 10.1038/s41405-022-00116-6

**Published:** 2022-08-01

**Authors:** Ghaith Alfakhry, Khattab Mustafa, M. Abdulhadi Alagha, Obada Zayegh, Hussam Milly, Feras Al Shwikani, Issam Jamous

**Affiliations:** 1 Education Quality and Scientific Research Office, Al-Sham Private University, Damascus, Syria; 2 Faculty of Dentistry, Damascus University, Damascus, Syria; 3 Program of Medical Education, Syrian Virtual University, Damascus, Syria; 4 Department of Restorative Dentistry, Faculty of Dentistry, Damascus University, Damascus, Syria; 5 Department of Orthopedics, Institute of Global Health Innovation, Imperial College London, London, UK; 6 College of Medicine and Health, University of Exeter, Exeter, UK; 7 Department of Fixed Prosthodontics, Faculty of Dentistry, Damascus University, Damascus, Syria

**Keywords:** Dental clinical teaching, Restorative dentistry

## Abstract

**Objectives:**

The study was conducted to investigate whether peer-assessment among dental students at the clinical stage can be fostered and become closer to that of experienced faculty assessors.

**Methods:**

A prospective pilot study was conducted in 2021 at the Faculty of Dentistry, Damascus University. Sixteen 5th year clinical students volunteered to participate in the study. A modified version of the validated Peer Direct Observation of Procedural Skills (Peer-DOPS) assessment form was used together with a grading rubric. Participants undertook peer-assessment on their colleagues across three encounters. The difference between peers and faculty assessment was the main variable.

**Results:**

The mean difference between peers and faculty assessment decreased after each encounter with a significant difference and a medium effect size between the first and third encounters (*p* = 0.016, d = 0.67). Peer-assessment was significantly higher than faculty, however, the overestimation declined with each encounter reducing the difference between peer- and faculty assessment. Peers’ perception of the educational benefit of conducting assessment was overwhelmingly positive, reporting improvements in their own performance.

**Conclusion:**

This pilot study provides preliminary evidence that dental students assessment ability of their peers can be fostered and become closer to that of experienced faculty assessment with practice and assessment-specific instruction.

## Introduction

There is an upsurge of interest in pursuing robust assessment methods of educational value [1]. The shift towards a partnership approach in clinical education, where learners play an active role in the learning process [2] begs the need to encourage student-centeredness through using peer-assessment methods [2, 3]. By definition, “ Peer-assessment is an arrangement for learners to consider and specify the level, value, or quality of a product or performance of other equal-status learners.” [4] In dental education, peer-assessment has shown promising educational benefits, primarily in academic achievement and life-long learning skills [5, 6].

Several studies in dental education have reported that exercising peer-tutoring and assessment can be advantageous to the assessors, the assessees, as well as the academic institutions. From a peer-assessor lens, evidence showed that peer-assessment could improve academic achievement, interpersonal, and lifelong-learning skills [7–9]. For the assessee, peer assessment is thought to provide a powerful learning tool that can foster peer-assisted reflection and learning [10, 11], as well as being less stressful for trainees in comparison to staff assessment [11]. At an academic institutional level, peer-tutoring is cost-effective, providing similar levels of educational attainments and equipping peer-tutors with a valuable pedagogical experience [8].

Peer-assessment has been piloted in dental education in both pre-clinical and clinical stages. However, the majority of studies were conducted in preclinical settings. In restorative dentistry, one study presented evidence of peers’ ability to assess and detect trainees’ improvement across various domains [5]. In another study [12], peer-assessment of pre-clinical restorative skills was not significantly different from experienced assessors. In maxillofacial surgery, a study investigating peer- and self-assessment reliability using a structured assessment tool showed that peer-assessors were more reliable than self-assessors in assessing surgical skills. The study showed no significant difference between peer-assessment and faculty assessment with a moderate correlation using checklist grading and a strong correlation in the global rating scale [13]. Likewise, another study [14] showed that peer-assessors can be effective especially if efforts are made to calibrate peer-assessors. However, the authors found a significant difference between faculty and peer-assessors in different areas of assessment, a finding echoed by two other studies [15, 16]. A recent meta-analysis [17] highlighted that peer-assessors in medical fields have a lower tendency to agree with tutor’s assessment suggesting that peers should assess various dimensions in addition to providing an overall grade. Familiarizing peers with the assessment criteria was suggested as a key element for the successful implementation of this method [17].

In order to bridge the gap in clinical assessment between peers and faculty of dental students, we developed and piloted a peer-assessment protocol, adapted to the validated Direct Observation of Procedural Skills (DOPS) form, at Damascus University Faculty of Dentistry. This study sought to investigate whether the difference between peer- and faculty assessment of clinical performance among undergraduate dental students can be reduced through deliberate training in three clinical encounters. A secondary objective was to investigate students’ perception regarding the educational benefits of peer-assessment. The authors hypothesised that there is no significant change in the mean difference between peer- and faculty assessment in the first and the last encounter.

## Methods

### Study design

This is a prospective pilot study that has been conducted at the Department of Restorative Dentistry at Damascus University Faculty of Dentistry in Syria. The research project was undertaken during the clinical training of students in restorative dentistry from the beginning of the second semester in late March 2021 until the end of the semester in late June 2021.

### Participants and settings

The dentistry programme at Damascus University is comprised of 5 years of training; three pre-clinical and two clinical years. In the clinical stage, students engage in treating patients in authentic work settings. This research was conducted during the course of clinical training of 5^th^ year dental students at the Department of Restorative Dentistry. Students were invited to participate in the study using an online survey. The recruitment survey was designed to screen students for their fitness and motivation to participate in the study. It was posted on the official Facebook group for students; the survey contained questions related to the demographics (age, sex), personal details (full name, phone number, email) and asked students for their consent to take part in the study. Students also asked to elaborate on the reason behind their motivation to participate. The recruitment process included all students who are in their final year and excluded participants who were unable to provide informed consent. Sixteen students agreed to participate in the study, serving as a convenience sample. The sample age range was between 22 and 23; female students comprised 62% (*n* = 10) of the sample.

Prior to the clinical course, participating students were evaluated by their previous clinical supervisors as “novice” in performing restorative treatment for class I, II, III, IV and V cases. The clinical supervisors evaluated participants as “novice” based on the performance they observed in the previous clinical restorative course during students’ 4^th^ year. Each student was required to assess another 5^th^ year student colleague across three clinical encounters. Six clinical faculty members were assigned as assessors so that by the end there was a peer-assessment and a faculty assessment for each trainee. All participants assessed each other with the exception for 5 peer-assessors who assessed trainees acting only as assessees and did not perform peer-assessment. Nevertheless, the pairs-peer-assessor and the assessee-were the same in every encounter. All assessees consented to participate in the study. It was made explicit to students prior to the study that the scores given by their peers or the participating faculty did not affect their official grades in the module.

### Data collection

A validated workplace assessment tool was used, namely Direct Observation of Procedural Skills (DOPS) [5]. The assessment tool development and pretesting were reported in a previous publication [18]. The grading scale was 5-point criterion-referenced (1 = clear fail, 2 = borderline fail, 3 = borderline pass, 4 = clear pass, 5 = excellent). Students also had to refer to the items they considered as (a) areas of improvement or (b) areas of excellence by the number they were assigned in the evaluation form. Students had to determine three areas of strengths and three areas of improvement in each encounter. The range of restorative procedures was limited to amalgam or composite restorations classes I, II, III, IV, V. A grading rubric was used in an effort to render the assessment process more valid and reliable, while providing an opportunity for focused constructive feedback [19]. The assessment form is available in a previous published work [18]. The assessment form included fourteen items which addressed three domains: knowledge, skills, and attitude. The last item asked the assessor to give a global score for the trainee.

Faculty and peers were instructed on how to assess using the DOPS form and grading rubric in an orientation session. The orientation session lasted for 20 min as a full-description of the project and the grading rubric was already provided electronically to all stakeholders. During the orientation session, a trained instructor (GA) familiarized participants with the process of peer-assessment: observation, taking notes, statements in the peer-DOPS form, and what each assessment scale point indicate in relation to the respective statement. The intersection between each assessment statement and grading scale, for example: “clear pass”, was defined and illustrated in a grading rubric prior to the study (Appendix I). The grading rubric design and pretesting were illustrated in another study [18]. As for the peer-assessors, they were included in a peer-assessment feedforward sessions that took place after each assessment session. During these sessions, basic principles of assessment were illustrated along with the relevant criteria; common mistakes in assessment were also highlighted. The 16 selected peer-assessors worked in pairs with their assessee. Peer-assessment and feedback were provided to the assessees immediately after the clinical encounter. After each encounter, the faculty, peers, and trainees conducted a feedback session in which performance issues were addressed as well as the discrepancy between peer- and faculty assessment. Peer-assessors were provided with the grading rubric and were instructed to use it in their assessment. The peer-assessment encounters occurred every week until all participants finished three Peer-DOPS encounters. The peer-DOPS lasted the entire clinical encounter which lasted on average between one hour thirty minutes and 2 h. Feedback delivery was moderated by the clinical supervisor and it rarely exceeded 5 min.

Peer-assessment scores were compared to scores given by the faculty. The mean difference between peers and faculty scores in all fourteen items was considered as the main variable. Peers’ responses on the two items in the feedback section which asked them to determine three areas of improvement and three areas of excellence were compared with that of the faculty. If the three points of the peer-assessor and the faculty were in total agreement, the peer score was 3 (total agreement), if the points made by the peer were totally different, the peer score was 0 (total disagreement). This way, peers’ ability to highlight strengths and areas of improvement was quantified.

The educational benefit of this peer-assessment was investigated using a post-course questionnaire. Students were asked about their attitudes on a 5-point Likert Scale statement and were also asked in an open-ended question about what they learnt from conducting peer-assessment. Questions and responses were recorded in Arabic and then translated to English by qualified translators.

### Data analysis

Normality was tested using Shapiro-Wilk test. Thereafter, Paired *t*-test was conducted to compare means of peer-assessment and faculty assessment in each encounter. The mean difference between faculty and peer assessment between the first and the third encounter was analyzed using the Paired *t*-test, and the effect size was measured using Cohen’s d. Wilcoxon signed-rank test was used to compare the number of areas of excellence and improvement peers selected and matched that of the faculty between the first and the last encounter. Thematic analysis was used to analyze students’ responses on the open-ended question in the post-course questionnaire.

Data processing was done on Google Sheets, and statistical analysis was conducted using IBM SPSS Statistics for Windows, version 26 (IBM Corp., Armonk, N.Y., USA). Cohen’s d was calculated using Microsoft Excel 2016. The online questionnaire was conducted via Google Forms. MAXQDA 2020 was used to code and analyze students’ responses.

## Results

Table 1 shows the difference between peer- and faculty assessment in the first, second, and third encounter. The difference is significant in all encounters; however, it varied in value. The mean difference consistently dropped after each encounter reaching less than 50% of its value in the first encounter with a significant difference between the first and third encounter and a medium effect size (*p* = 0.016, d = 0.67). Faculty assessment remained stable across the three encounters with no significant difference between their mean scores in the first and third encounter. In contrast, peer-assessment decreased consistently after each encounter; a significant difference in peer-assessment was detected between the first and the last encounter with a large effect size (*p* = 0.005, d = 0.83) (Table 2).

**Table 1:** The mean difference between faculty and peer-assessment in each of the 3 encounters along with peer-assessment and faculty assessment.

	*n*	1st encounter Mean (SD)	2nd encounter Mean (SD)	3rd encounter Mean (SD)
Mean difference between peers and faculty	16	0.76*** ± 0.45	0.57** ± 0.58	0.37* ± 0.58
Faculty assessment	16	2.94 ± 0.44	2.88 ± 0.43	2.84 ± 0.36
Peer-assessment	16	3.71 ± 0.43	3.45 ± 0.45	3.22 ± 0.59

*SD* Standard deviation which is at a 95% confidence interval, *p < 0.05, **p < 0.01, ***p < 0.001.

**Table 2:** Paired *t*-test comparing mean difference between peers and faculty in the first encounter and the last (3rd) encounter.

Pairs	*n*.	MD (95% CI)	*p*-value	Effect size (Cohen’s d)
Mean difference between peers and faculty 1–3	16	0.39 (0.08 to 0.70)	0.016	0.67
Faculty assessment 1–3	16	0.09 ± 0.44	0.42	0.20
Peer-assessment 1–3	16	0.48 ± 0.58	0.005	0.83

*MD* mean difference, *SD* Standard deviation which is at a 95% confidence interval.

**Fig. 1:**
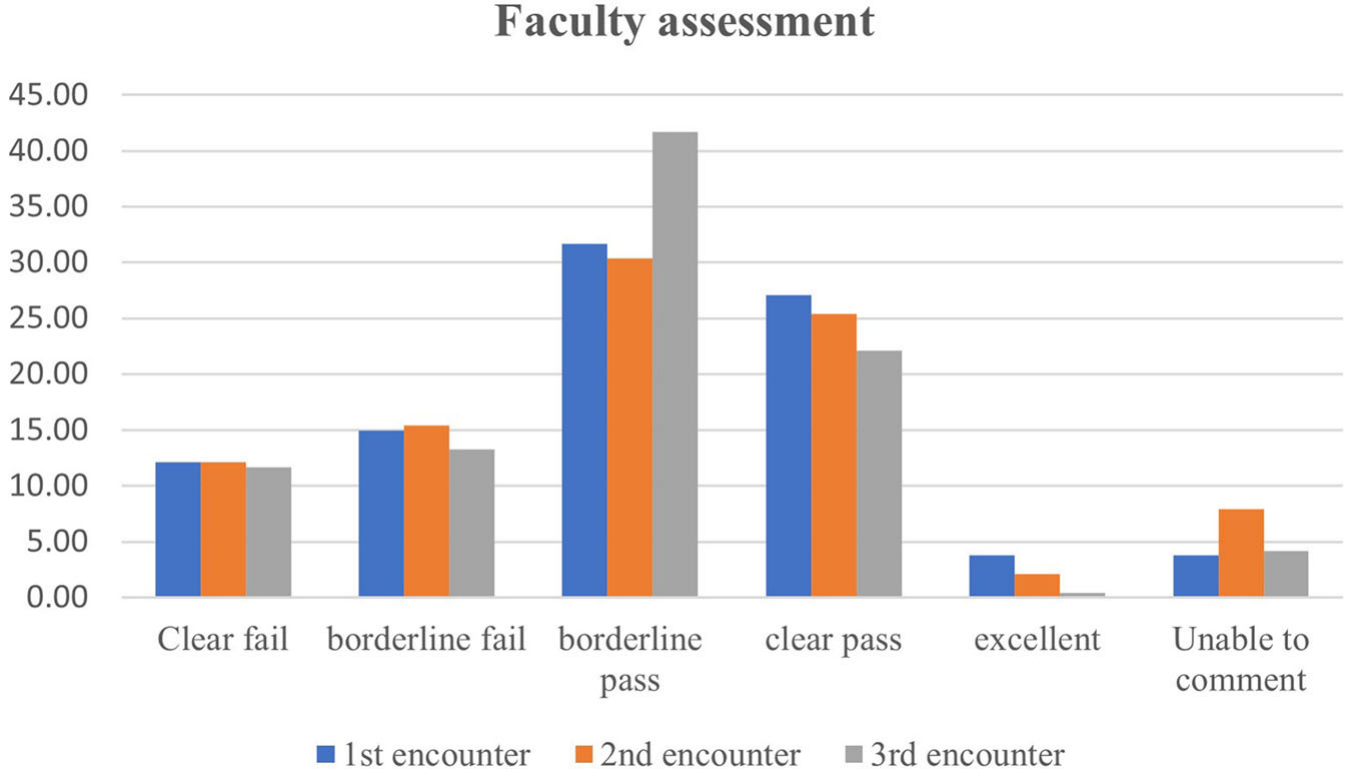
Faculty assessment variation on the scale level. A bar chart showing the percentage of each of the 5-point assessment scale given by clinical faculty in the first, second and third encounter.

**Fig. 2:**
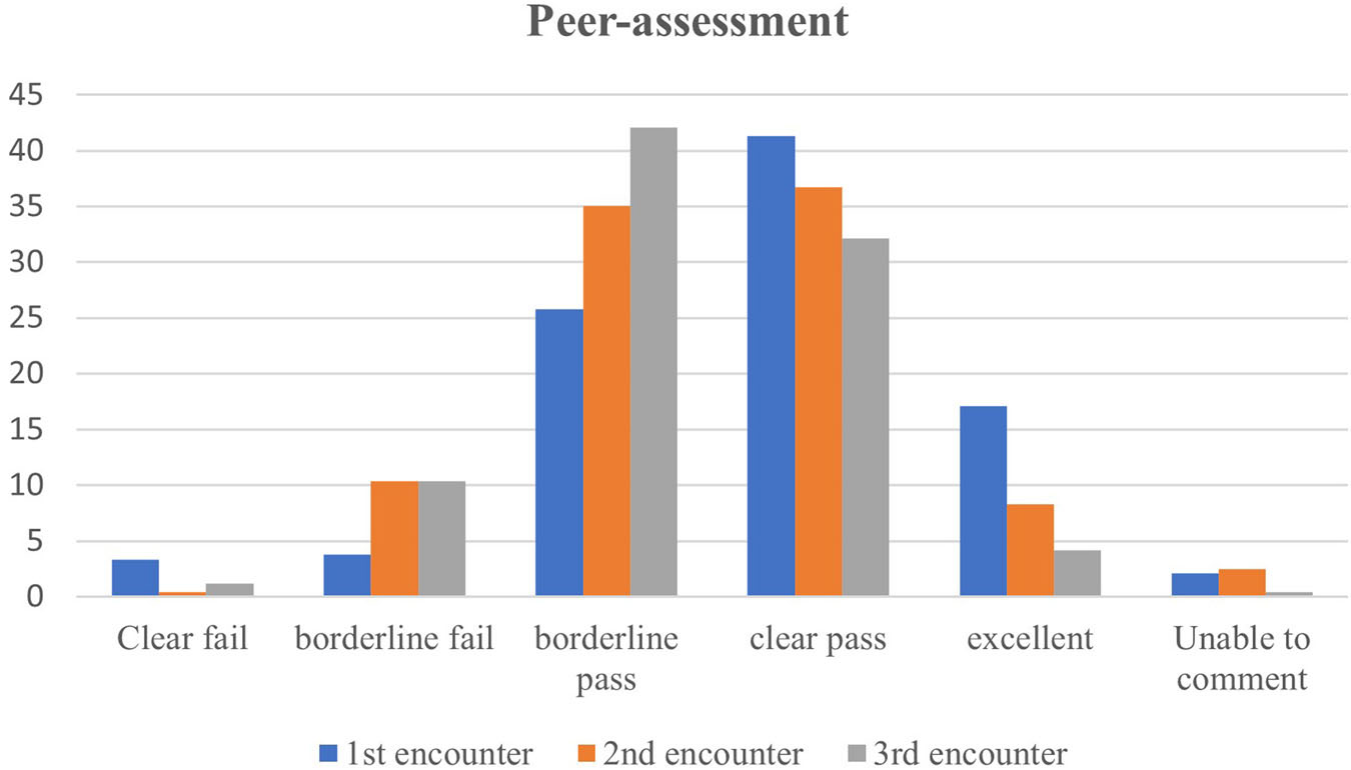
Peer-assessment variation on the scale level. A bar chart showing the percentage of each of the 5-point assessment scale given by peers in the first, second and third encounter.

Figures 1 and 2 illustrate the percentage of ‘clear fail’, ‘borderline fail’, ‘borderline pass’, ‘clear pass’ and ‘excellent’ evaluations given by faculty and peers, respectively. Faculty gave relatively close percentage of each rating across the three encounters in exception for the borderline pass which increased 10 percent in the third encounter. On the other hand, peers gave less and less evaluations as ‘excellent’ and ‘clear pass’ after each encounter while giving more evaluations as ‘borderline pass’ and ‘borderline fail’.

Table 3 shows the mean difference between faculty and peer scores in each performance domain and dimension. The mean difference between faculty and peers decreased in most performance dimensions through the three clinical encounters. In the 1st, 2nd,5th,7th and 10th, performance items, there was no significant difference between peer- and faculty assessment in the third encounter in contrast to the first encounter. The mean difference was the lowest and relatively stable in items concerning ‘Organization, efficiency and time management’, ‘Chair, patient and dentist’s position’. In the restoration evaluation item, mean difference was equal to zero in the second encounter indicating that peers and faculty scores had identical means (*p* = 1). In items no. 3, 4, 11 in the attitude domain as per Table 3, the significant difference between peers and faculty continued across the three encounters. The mean difference between peers and faculty assessment in the overall performance evaluation no. 14 remained comparatively small and stable across encounters.

**Table 3:** Paired *t*-test showing the mean difference between peers and faculty in each assessment criteria in each encounter.

	1st encounter	2nd encounter	3rd encounter
Assessment criteria	MD (SD)	MD (SD)	MD (SD)
1. Clinical assessment, diagnosis and treatment plan	1.37*** ± 1.08	0.20 ± 1.20	0.13 ± 0.91
2. Demonstrates understanding of indications, dental materials and used technique	1.18*** ± 0.91	0.33 ± 1.04	0.25 ± 1.00
3. Obtaining patient consent after explaining the procedure and possible complications	1.18* ± 0.42	0.86* ± 1.12	0.81* ± 1.42
4. Pre-procedural preparation	1.06 ± 0.52	1.7*** ± 1.3	1.36** ± 1.36
5. Pain, anxiety management and communication skills	0.81** ± 0.24	0.62 ± 1.5	0.26 ± 1.43
6. Chair, patient and dentist’s position	−0.06 ± 0.28	0.12 ± 1.08	−0.06 ± 1.06
7. Preparation for the restoration	1.06** ± 0.29	0.50 ± 1.45	0.33 ± 1.04
8. Restoration	0.21 ± 0.28	0 ± 1.15 (*p* > 0.999)	0.33 ± 0.81
9. Infection control and safe disposal of biohazard materials and sharp tools	0.5 ± 0.35	0.81 ± 179	0.25 ± 1.18
10. Seeking help when appropriate.	0.62** ± 0.20	0.53 ± 1.45	0.12 ± 1.20
11. Patient education	1.6** ± 0.42	1.84*** ± 1.46	1.06*** ± 0.88
12. Organization, efficiency and time management	0.2 ± 0.38	−0.13 ± 1.12	0.12 ± 1.40
13. Professionalism	0.37 ± 0.23	0.13 ± 0.83	0.18 ± 0.83
14. Overall performance assessment	0.33 ± 0.36	0.35 ± 0.84	0.37 ± 0.71

*MD* Mean difference, *SD* Standard deviation which is at a 95% confidence interval. *p < 0.05, **p < 0.01, ***p < 0.001.

Peers’ ability to highlight areas of improvement defined by the experienced faculty varied from session to session. The peers’ group were able to identify 15, 17, 10 areas of improvement in the first, second and third encounter, respectively. In contrast, peers’ ability to identify areas of strengths improved consistently over the sessions; the sum of strengths that were identified correctly by peers were 5, 11, 12, respectively in each session. There was no significant difference between the sum of correctly identified strengths in the first and the third session (*p* = 0.07) according to the related-samples Wilcoxon signed rank test.

In the post-course questionnaire, 14 out of 16 students responded. Participants were asked if they learnt from conducting peer-assessment on a 5-point Likert scale ranging from totally disagree to totally agree; 10 agreed, 1 disagreed and 3 stayed neutral. Students commented on what they learnt from doing peer-assessment. Their responses were analyzed and three distinct themes emerged: improvement in performance, self-assessment, and feedback delivery. The themes and students’ selected responses are shown in Table 4.

**Table 4:** Selected comments that pertain to each theme extracted from students’ responses on the post-course questionnaire item “What did you learn from peer-assessment?”.

Themes	Students’ comments
Improvement in performance	*“I started focusing more when I treat patients to avoid making my colleague’s mistakes which I learnt from”* (study participant) *“We observed more cases, learnt from the assessment and notes provided by the supervisors which increased our clinical knowledge”* (study participant) *“I learnt from identifying my colleague’s areas of improvement and his areas of strengths.”* (study participant) This was also echoed by two other students.
Self-assessment	*“I learnt to be aware of performance issues that I used to consider ‘correct’ when I make but not ok when I see my colleague makes.”* (study participant) *“I became more able to identify my own mistakes that I used to make without being aware.”* (study participant) This response was echoed by another student.
Feedback delivery	*“I learnt to objectively criticize my peer”* (study participant) *“We learnt to accept each other’s feedback with complete transparency.”* (study participant)

## Discussion

This is the first peer-DOPS implementation at Damascus University Faculty of Dentistry. This study aimed to investigate whether the gap between peer-assessment and expert faculty assessment can be bridged through focused instruction and practice in assessment. The findings revealed a significant reduction in the gap between peers’ and faculty in the third encounter in comparison to the first encounter. Peer-assessment was an overestimation of trainees’ performance, which declined with each encounter thus reducing the difference with faculty assessment. Even though trainees’ performance remained relatively stable according to faculty assessment across the three encounters, peer-assessment changed significantly by the third encounter with a large effect size. Peers expressed an overwhelmingly positive attitude of the educational benefit of conducting peer-assessment; peer-assessors reported improvement in their own performance, feedback delivery and self-assessment ability.

Peer-assessment accuracy could vary according to a number of factors, including: context, course level, type of product or performance, evaluation criteria clarity and assessment training and support [4]. Precision in the assessment of practice can be more difficult to achieve than the assessment of academic products [4]. Assessment of clinical performance is complex and multidimensional, encompassing cognitive, psychomotor and non-clinical skills [20]. Unlike knowledge, which is a relatively stable entity by itself [21], performance varies considerably and inevitably in different occasions and settings, making accurate assessment even more challenging [20]. The majority of peer-assessment studies in the field of operative/restorative dentistry are preclinical; these studies showed mixed results in terms of peer-assessment accuracy in comparison to experienced assessors [12, 22]. In this study, peer-assessors consistently overcalled trainees’ performance giving significantly higher scores than faculty and this is concurrent with previous study findings [22, 23]. The current findings showed that the reduction of the gap between peer- and faculty assessors varied across performance domains.; for instance, a significant difference persisted in the ‘patient education’ domain across all three encounters. In contrast, areas of assessment pertaining to technical skills such as ‘restoration’, ‘chair and patient position’ showed a small mean difference between peers and faculty. It’s beyond the scope of this study to explain the reason for such discrepancy in assessing different performance areas; however, a previous preclinical study showed that the difference between peer-assessor and expert assessors also varied according to the assessed domain [14]. Students positive attitude towards peer-assessment has been reported in previous studies [5, 10, 24]. The impact of conducting peer-assessment on students assessors performance, reflection and interpersonal skills has also been previously reported [7].

Despite the consistent overcalling of performance by peers, the degree of overestimation decreased significantly, and the gap between peer-assessment and trained faculty narrowed significantly in the third encounter when compared to the first encounter. A possible reason for this decline is that peer-assessors contrasted their assessment with that of faculty after each encounter during the feedback session. Faculty highlighted participants’ issues in assessment including the overcalling of trainees’ performance. When a participant overestimated a trainee performance, the reason behind this was explored, the assessment criteria was re-illustrated and the observed performance aspects that merit a lower grading were shared with the participant. It was noticed that participants had problems in observing, recording and analyzing trainees’ performance. At the beginning, most participants failed to point out to specific observations which merit a specific grade. In time, participants were able to substantiate their grading with accurate observations. This result supports the notion that peer-assessment can be improved by providing peer-assessors with training, checklists, grading criteria and teacher support [4]. Another possible explanation is that peer-assessors could have become more used to faculty assessment and therefore concordance improved.

This study found that peers’ ability to highlight areas of strengths improved consistently after each encounter. However, their ability to identify areas of improvement were volatile. The exact reason for this is not clear. Nevertheless, the same finding was found in a previous study that used the exact measurement of identifying strengths and areas of improvement in restorative dentistry but measuring self-assessment instead of peer-assessment [18]. Some authors suggested that peer-assessment practices enhances self-assessment skills amongst students and therefore considered it a dimension of self-assessment [25]. This could explain the similar findings between this study and the self-assessment study [18]. Moreover, in comparison to the self-assessment study which used the same assessment training protocol, peer-assessors improvement in accuracy is very similar to the improvement observed in students’ self-assessment ability which corroborates the idea that peer-assessment and self-assessment are connected. This is further supported by the fact that the emerging themes from post-course questionnaire were similar to the themes extracted from the self-assessment study [18]. A comparison between these two studies revealed that both self-assessment and peer-assessment helped students improve their attention focusing, observation, clinical performance, reflection, and identifying oneself areas of excellence and improvement. In contrast to self-assessment, participants reported that peer-assessment helped them become more self-aware as redefining a performance aspect from ‘correct’ to ‘in need of improvement’. Further, peer-assessment provided students with more learning opportunities, helped them practice giving objective feedback and accept criticism from others.

The area of whether the gap between peer-assessment and experienced assessors in clinical dentistry can be reduced has been relatively unexplored, making this study a valuable addition to the dental education literature. This study started from the notion that orientation sessions may not be enough to improve peer-assessment accuracy [26, 27]. Therefore, a complete training protocol with a structured assessment form and a detailed grading rubric were developed to address the gap between peer-assessors and expert assessors. The findings of this study support the idea that peer-assessment is a learnable skill that can be harnessed through deliberate training and the use of structured assessment tool with clear criteria [4]. It could be useful to conduct peer-DOPS regularly during clinical restorative courses due to the educational benefits of peer-assessment as shown in the current study and previous research [7–11]. Clinical supervisors could carry out DOPS in conjunction with peer-DOPS three to six times per year as suggested by previous studies [28, 29].

Our study has few limitations; although inter- nor intra- -rater reliability between assessors were not evaluated prior to the study, the faculty were trained to assess using the grading rubric which according to previous studies can increase the validity and reliability of the used assessment method [19]. A control group was not applied so there might have been some factors that could have affected the results. Peer-assessment bias and friendship bias cannot be excluded although students were directly informed that the scores given by their peers or faculty in this pilot study would not affect their official scores in the clinical module. The nature of workplace assessment also presents numerous variables which could have affected the findings. Nonetheless, procedures, case difficulty, materials and allocated time were variables that were standardized among participants. The sample size and the limited number of encounters is also an area that limits the generalizability of the study findings. Future research needs to address these limitations and use a larger sample size, double-blinded designs across different institutions and over extended period of time.

## Conclusion

This prospective pilot study provides some evidence that the gap between peer-assessment and faculty assessment could be bridged through deliberate training and assessment-oriented feedback taking into account that a structured assessment method with a clearly defined criteria is used.

## Supplementary information

Appendix I
