# Supplementary material for: Peer-assessment ability of trainees in clinical restorative dentistry: can it be fostered?
Source: BDJ Open. 2022 Aug 1;8:22. doi: 10.1038/s41405-022-00116-6 (PMC9343389; doi:10.1038/s41405-022-00116-6)
Supplement: Supplementary file 1 — Appendix I [file 41405_2022_116_MOESM1_ESM.docx]

| **Item** | **1. Clear fail** | **2. Borderline fail** | **3. Borderline pass** | **4. Clear Pass** | **5. Excellent** |
| --- | --- | --- | --- | --- | --- |
| **1. Clinical assessment, diagnosis and treatment plan** | Unable to identify/conduct appropriate diagnostic tests | Able to identify/conduct appropriate diagnostic tests  Unable to identify a differential or definitive diagnosis | Requires assistance to establish a differential diagnosis and treatment plan based on the data collected | Requires minimum assistance to establish a differential diagnosis and treatment plan based on the data collected | Independently establishes a differential diagnosis and treatment plan based on the data collected |
| **2. Demonstrates understanding of indications, dental materials and used technique** | No knowledge at all | Have some knowledge of what needs to be done but missing some or all critical points (requires lots of help) | -Have acceptable knowledge of what needs to be done(requires some help) -Addresses all main steps  -Answers follow up questions fairly with some minor errors | Have a clear picture of what needs to be done (requires minimum help) -Answers follow up questions well and clearly with few errors | -Have an outstanding knowledge of the procedure and the steps (can work independently)  -Answers all follow up questions proficiently |
| **3. Obtaining patient consent after explaining the procedure and possible complications** | -Doesn’t explain anything to the patient or explains minimally. | -Explains to the patient partially, but doesn’t give the patient the chance to ask questions-his explanation is not easy to understand by the patient | -Explains the whole procedure fairly, allows the patient to ask questions. -Doesn’t address the patient concerns properly. | -Explains the whole procedure well, allows the patient to ask questions. -Addresses the patient concerns properly. -Fairly aware of these concerns | -Explains the whole procedure at a professional level, allows the patient to ask questions. -Addresses the patient concerns properly. -Well aware of these concerns |
| **4. Pre-procedural preparation** | -Unprepared, unaware of the steps and the procedure to satisfactorily meet the required standards | -Minimally prepared -Unclean, Untidy working place | -Acceptable preparation -Clean and tidy working place | -Good preparation  -Clean and tidy working place | -Outstanding preparation  -Clean and tidy working place, everything is placed strategically |
| **5. Pain and anxiety management and communication skills** | -Inappropriate pain control  -Ineffective communication: failure to establish functional rapport with patient  -Unaware or uninterested in patient's needs  -Displays closed-mindedness by resisting patient feedback. | -Minimally prepared, help needed with pain control  -Does not fully recognize or understand the interpersonal needs of the patient  -Challenged communication  -Does not seek feedback | -Acceptable pain control  -Demonstrates some compassion and respect for patient  -Recognizes some of the patients' needs in the context of their lives and their oral care  -Acceptable communication  -Seeks feedback | --Good pain control  -Demonstrates a good level of compassion and respect for patient,  -Clearly recognizes patients' needs in the context of their lives and their oral care  -Displays fair-mindedness and actively seeks feedback | -Outstanding pain control  -Demonstrates a high level of compassion and respect for patient,  -Clearly recognizes patients' needs in the context of their lives and their oral care  -Skilled and purposeful communication which demonstrates sensitivity to cultural diversity  -Displays fair-mindedness and actively seeks feedback |
| **6. Chair, patient and dentist’s position** | No skills or knowledge on how to manage | Poor skill (needs instructions) | Acceptable skill (needs minimum instruction) | Good skill (able to to work smoothly during the procedure) | Professional-like skill |
| **7. Preparation for the restoration (Amalgam)** | -Isolation and initial access (poor)  -Final access (over or under extended, adjacent tooth damage): poor  -Enamel support (fail) -Depth (fail) -Wall orientation: poor -Pathology Removal/ mechanical pulp exposure: Fail Poor: requires major modifications | -Isolation and initial access :poor  -Final access: poor  -Enamel support (pass) -Depth (pass) -Wall orientation: poor -Pathology Removal/ mechanical pulp exposure: pass Poor: requires major modifications | -Isolation and initial access: fair -Final access: fair -Enamel support: pass -Depth: (pass) -wall orientation: fair -Pathology Removal/ mechanical pulp exposure: pass fair: requires some modification | -Isolation and initial access: good -Final access: good -Enamel support: pass -Depth: (pass) -wall orientation: good -Pathology Removal/ mechanical -pulp exposure: pass good: requires minimum modification | -Isolation and initial access: excellent -Final access: excellent -Enamel support: pass -Depth: (pass) -wall orientation: excellent -Pathology Removal/ mechanical pulp exposure: pass Excellent: requires no modification |
| **7. Preparation for the restoration (composite)** | -Isolation and initial access (poor)  -Final access (over or under extended, adjacent tooth damage): poor -Pathology Removal/ mechanical pulp exposure: Fail | -Isolation and initial access :poor  -Final access: poor  -Pathology Removal/ mechanical pulp exposure: pass | -Isolation and initial access: fair Final access: fair -Pathology Removal/ mechanical -pulp exposure: pass fair: requires some modification | -Isolation and initial access: good -Final access: good -Pathology Removal/ mechanical pulp exposure: pass good: requires minimum modification | -Isolation and initial access: excellent -Final access: excellent -Pathology Removal/ mechanical pulp exposure: pass Excellent: requires no modification |
| **8. Restoration** | -Matrix, Wedge(s), Base, Liner: (poor)  -Cavosurface: Excess/Submargination (poor)  -Restoration Surface/Shade Smooth/Rough/Insufficient Finish(poor)  -Axial Anatomy Facial, lingual, proximal contours (including contact) (poor)  -Occlusal Anatomy (do not grade for Class V) Fossa, grooves, marginal ridges, cusp placement & occlusion (poor) | -Matrix, Wedge(s), Base, Liner: (fair)  -Cavosurface: Excess/Submargination (fair)  -Restoration Surface/Shade Smooth/Rough/Insufficient Finish(poor)  -Axial Anatomy Facial, lingual, proximal contours (including contact) (fair)  -Occlusal Anatomy Fossa, grooves, marginal ridges, cusp placement & occlusion (poor) | -Matrix, Wedge(s), Base, Liner: (fair)  Cavosurface: Excess/Submargination (fair)  -Restoration Surface/Shade Smooth/Rough/Insufficient Finish(fair)  -Axial Anatomy Facial, lingual, proximal contours (including contact) (fair)  -Occlusal Anatomy Fossa, grooves, marginal ridges, cusp placement & occlusion (fair) | -Matrix, Wedge(s), Base, Liner: (good)  -Cavosurface: Excess/Submargination (good)  -Restoration Surface/Shade Smooth/Rough/Insufficient Finish(good)  -Axial Anatomy Facial, lingual, proximal contours (including contact) (good)  -Occlusal Anatomy Fossa, grooves, marginal ridges, cusp placement & occlusion (good) | -Matrix, Wedge(s), Base, Liner: (excellent)  -Cavosurface: Excess/Submargination (excellent)  -Restoration Surface/Shade Smooth/Rough/Insufficient Finish(excellent)  -Axial Anatomy Facial, lingual, proximal contours (including contact) (excellent)  -Occlusal Anatomy Fossa, grooves, marginal ridges, cusp placement & occlusion (excellent) |
| **9. Infection control and safe disposal of biohazard materials and sharp tools.** | -Cannot demonstrate any infection control techniques nor safe disposal of sharps | -Demonstrates some proper infection control technique and safe disposal, but requires lots of assistance and reminders. | -Demonstrates acceptable infection control technique and safe disposal with minimum assistance | -Demonstrates proper infection control and safe disposal of sharps  minimum instruction is needed | -demonstrates evidence-based infection control and safe disposal of sharps  (done independently) |
| **10. Seeking help when appropriate** | -Doesn’t ask for assistance when the procedure is out of his scope of competence | -Seeks help just to get the procedure done, his questions are irrelevant | -Seeks help to learn and manage the case.  -His questions are somewhat mindful and purposeful (has a sense of what he needs to know) | -Seeks more information appropriately and asks insightful relevant questions that addresses what needs to be done | -Seeks more information appropriately and asks insightful relevant questions that addresses not just what needs to be done but to improve the treatment delivered and improve performance |
| **11. Patient education** | Not done | Done poorly | Done fairly | Done well | Professional level of patient education with consideration of the measures that the patient can do to preserve oral health |
| **12. Organization/ efficiency and time management** | -Acceptable standard was not met Time was improperly managed, not finished on time and/or the patient must return to complete procedure. The paperwork and grading may have to be done after clinical hours. | -Acceptable standard was not totally met with major assistance Student finishes a bit late with the patient  -the paperwork and grading have to be done after clinical hours | -Acceptable preparation and time management -Student finishes on time ( treatment and paperwork) - no time is spared for cleaning and packing dental tools and materials | -Student is prepared to perform the procedure with minimum assistance   -Student finishes on time ( treatment and paperwork). -Have enough time to clean and pack dental tools and materials | -Outstanding preparation, record management, time utilization.  -Independently  -Student finishes on time ( treatment and paperwork). -Have enough time to clean and pack dental tools and materials |
| **13. Professionalism** | Shows any ethical misconduct | -Adheres to code of conduct but poorly  -Respecting patients: poor  -Trustworthiness: poor  -Professional attitude poor | -Adheres to code of conduct and ethical code  -Respects patients  -Trustworthiness: fair  -Fair professional attitude | -adhering to ethical and legal standards, trustworthy, altruistic, shows integrity, caring, community focused Shows professional attitude with peers, faculty and the patient | adhering to ethical and legal standards, trustworthy, Altruistic, showing integrity, caring, community focused, and committed to excellence, high professional attitudes with peers, faculty and patient |
